# Supplementary figures and images for: After the bite: bacterial transmission from grey seals (Halichoerus grypus) to harbour porpoises (Phocoena phocoena)
Source: R Soc Open Sci. 2020 May 13;7(5):192079. doi: 10.1098/rsos.192079 (PMC7277243; doi:10.1098/rsos.192079)

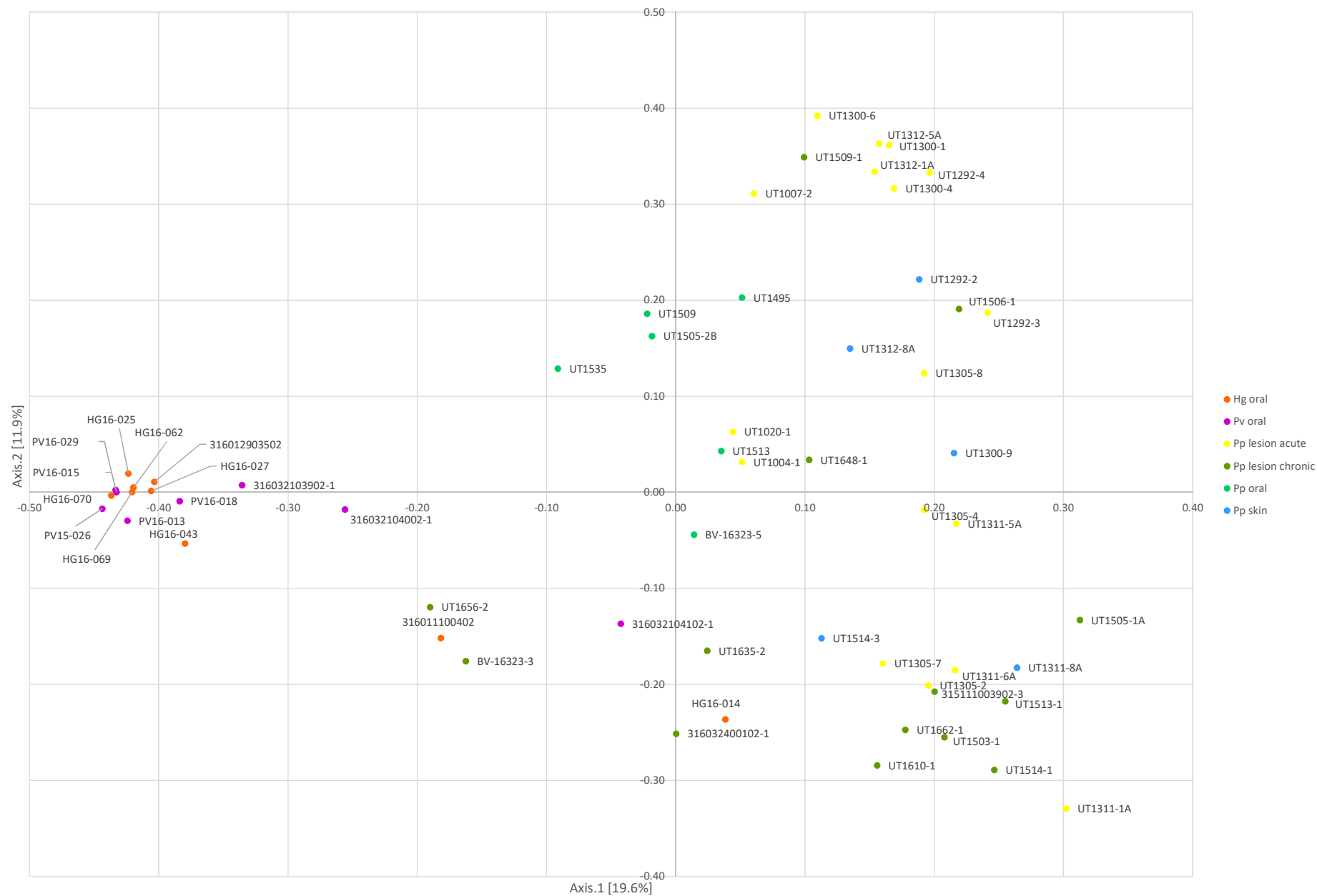

Supplement: Figure S1 [file RSOS192079supp1.pdf]

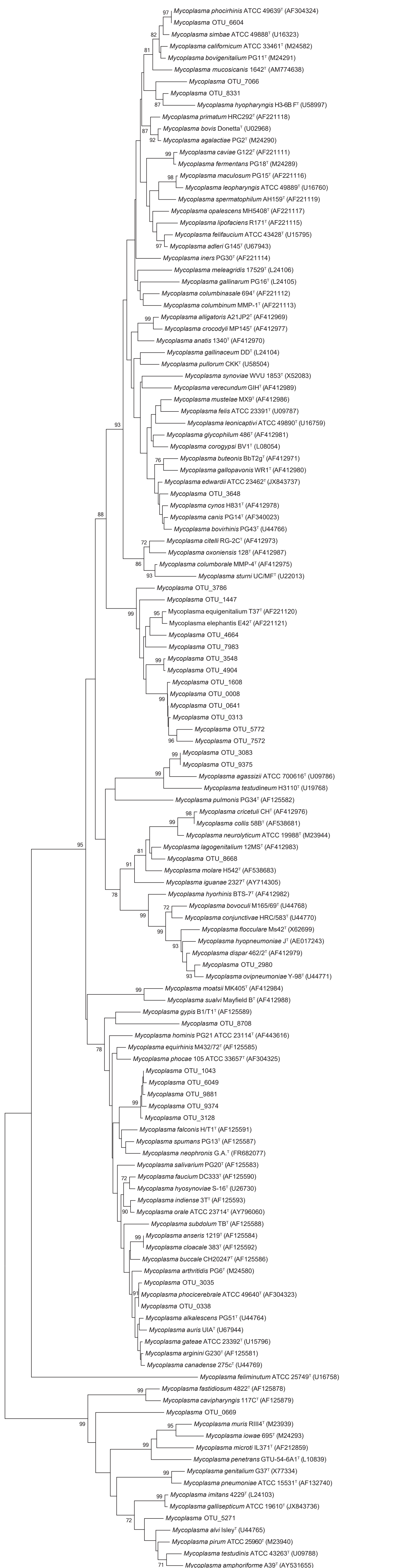

Supplement: Figure S2 [file RSOS192079supp2.pdf]
